# Supplementary material for: Spironolactone, a Classic Potassium-Sparing Diuretic, Reduces Survivin Expression and Chemosensitizes Cancer Cells to Non-DNA-Damaging Anticancer Drugs
Source: Cancers (Basel). 2019 Oct 14;11(10):1550. doi: 10.3390/cancers11101550 (PMC6826935; doi:10.3390/cancers11101550)
Supplement: Supplementary file 1 [file cancers-11-01550-s001.pdf]

cancers

Supplementary Materials

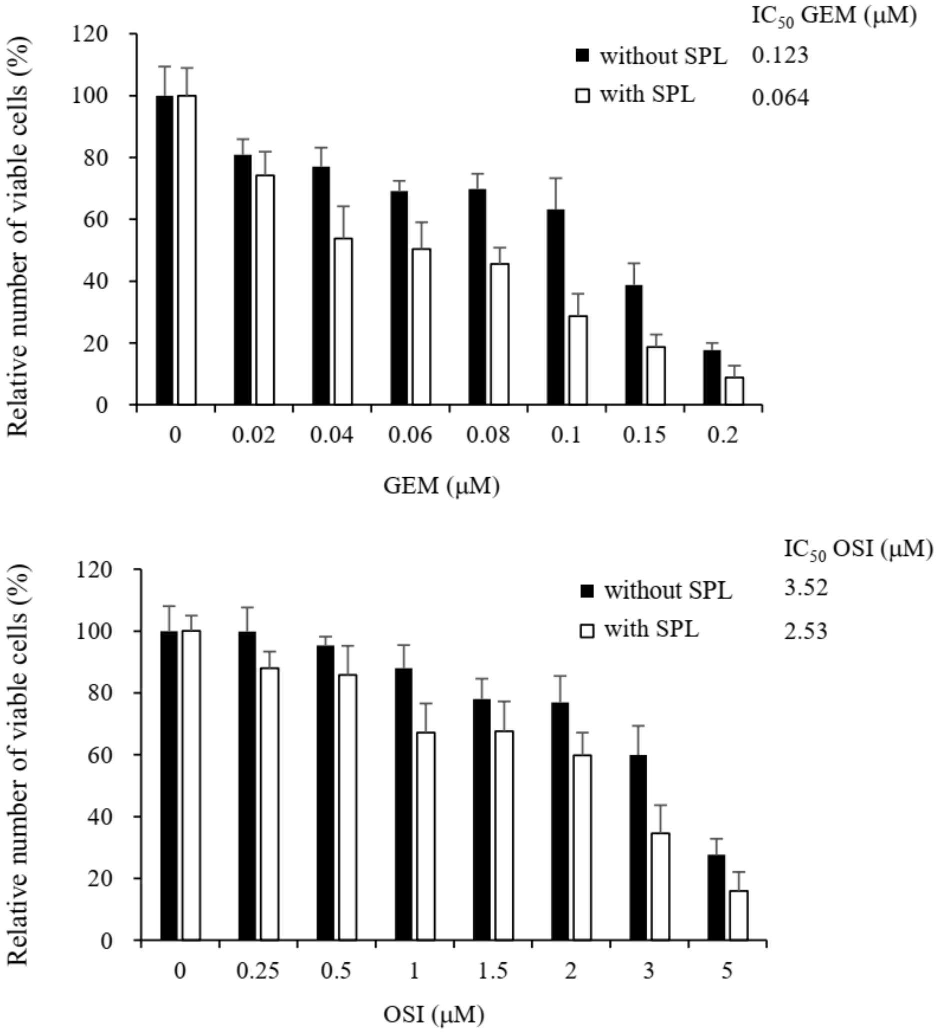

Figure S1. Effects of spironolactone on the viability of A549 cells treated with gemcitabine and osimertinib. A549 cells were cultured with various concentrations of gemcitabine (GEM) or osimertinib (OSI) in the absence (closed bars) or presence (open bars) of 25 µM spironolactone (SPL) for three days. The number of viable cells was quantified using Trypan blue. Values in the graph are expressed as relative to controls (i.e., drug concentration = 0 µM) and represent means + SD from triplicate samples.

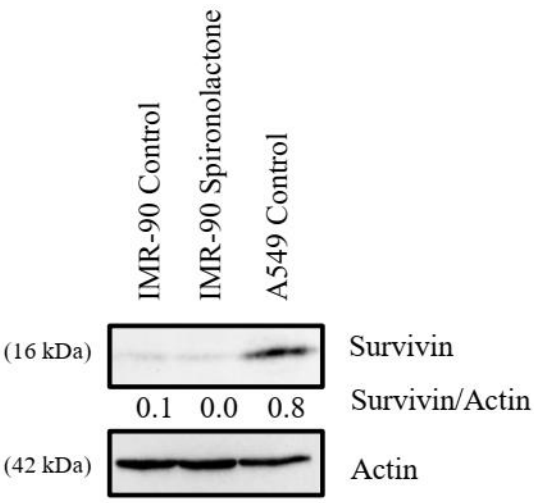

Figure S2. IMR90 cells express markedly lower levels of survivin than cancer cells. IMR90 cells were cultured in the absence (control) or presence (spironolactone) of 25 μM spironolactone for three days and subjected to an immunoblot analysis of survivin with a sample of untreated A549 cells.

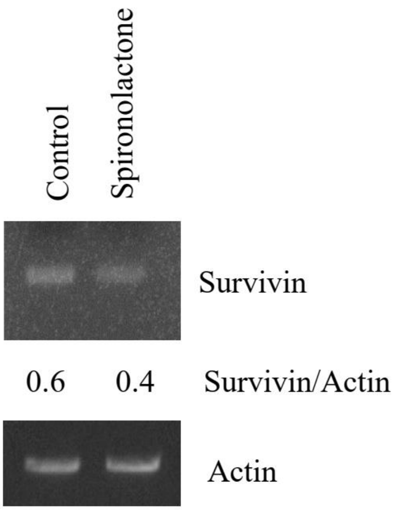

Figure S3. Spironolactone decreases survivin mRNA levels to a lesser extent. A549 cells treated with or without 25 μM spironolactone for three days were subjected to a reverse transcription-PCR analysis of survivin.

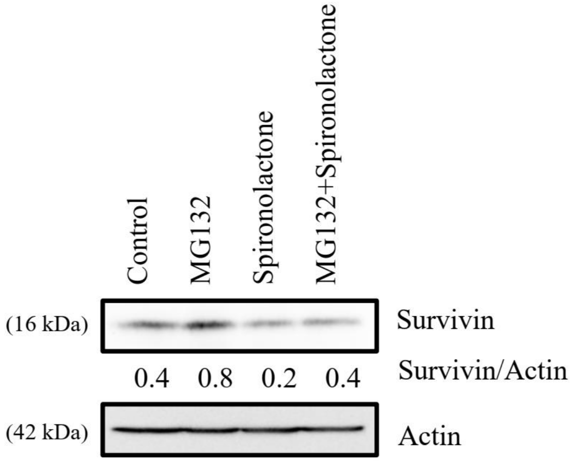

Figure S4. MG132 partially restores spironolactone-induced reductions in survivin. A549 cells treated or not treated with 25 μM spironolactone for three days and subsequently treated or not treated with 10 μM MG132 for 8 h were subjected to an immunoblot analysis of survivin.

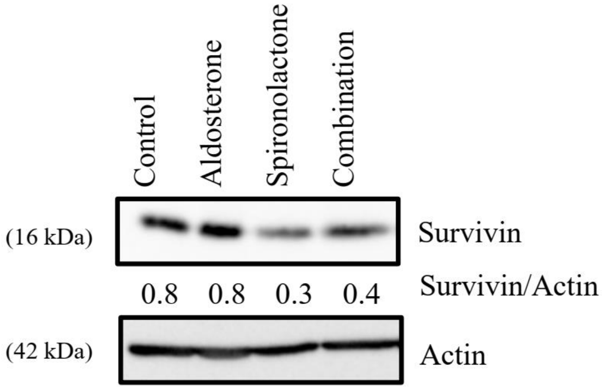

Figure S5. Effects of aldosterone on survivin reductions by spironolactone. PANC-1 CSLC cells were treated with aldosterone (10 μM), spironolactone (50 μM), or their combination for three days and then subjected to an immunoblot analysis for survivin.

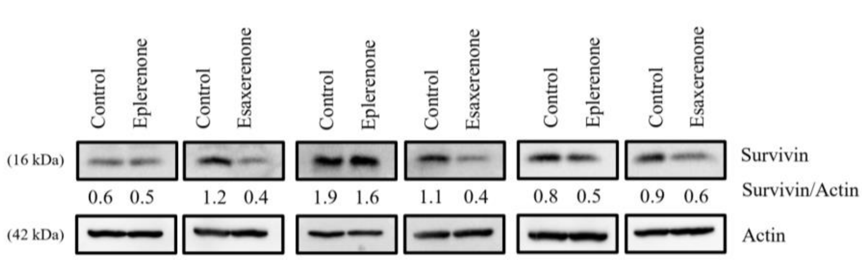

Figure S6. Effects of next-generation potassium-sparing diuretic reagents, eplerenone and esaxerenone, on the expression of survivin. The indicated cells were treated with eplerenone (50 μM) or esaxerenone (10 μM) for three days and subjected to an immunoblot analysis of survivin.

Figure S7

【Figure 4A】

A549 PANC-1 PC-9 PC-9-OR

Survivin

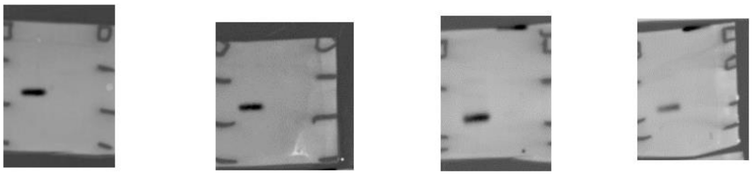

A549 PANC-1 PC-9 PC-9-OR

Actin

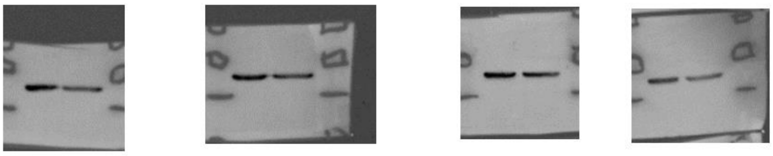

【Figure 4B】

A549 CSLC PANC-1 CSLC

Survivin

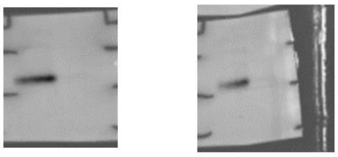

A549 PANC-1

Actin

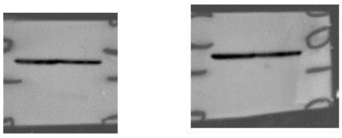

Figure 4C

YM155 siSurvivin

Survivin

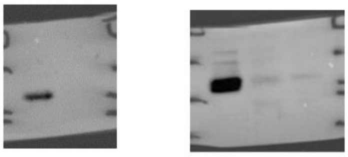

YM155 siSurvivin

Actin

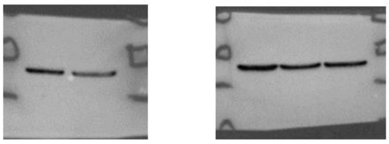

【Figure 5B】

Survivin

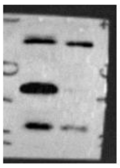

Actin

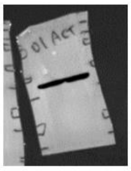

Figure S7 *Cont.*

Figure S7 (Continued 1)

【Figure S2】

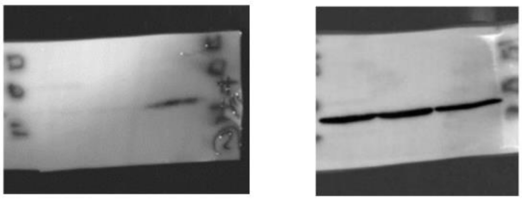

【Figure S3】

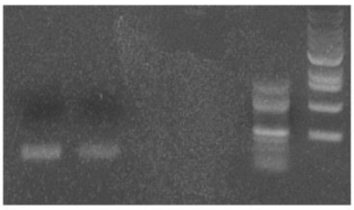

Survivin (330 bp)

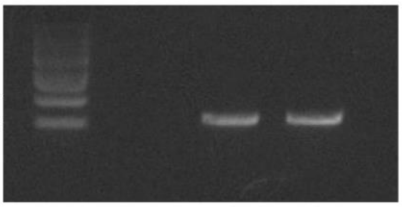

Actin (540 bp)

【Figure S4】

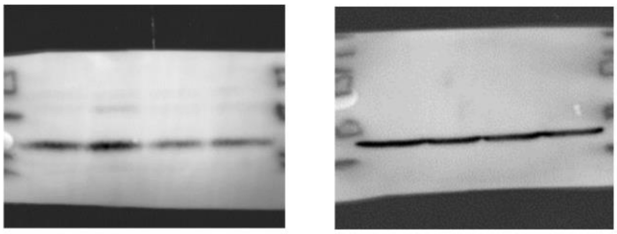

Figure S7 *Cont.*

Figure S7 (Continued 2)

【Figure S5】

Survivin Actin

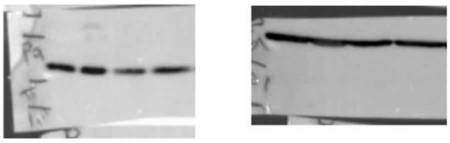

【Figure S6】

A549 CSLC PANC-1 CSLC GS-Y01

Survivin Survivin Survivin

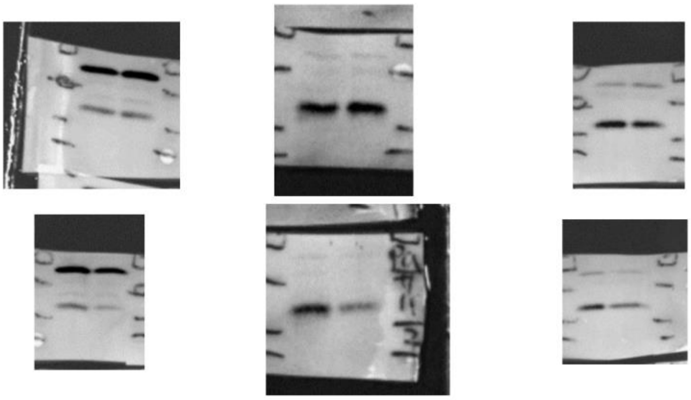

Eplerenone

Esaxerenone

A549 CSLC PANC-1 CSLC GS-Y01

Actin Actin Actin

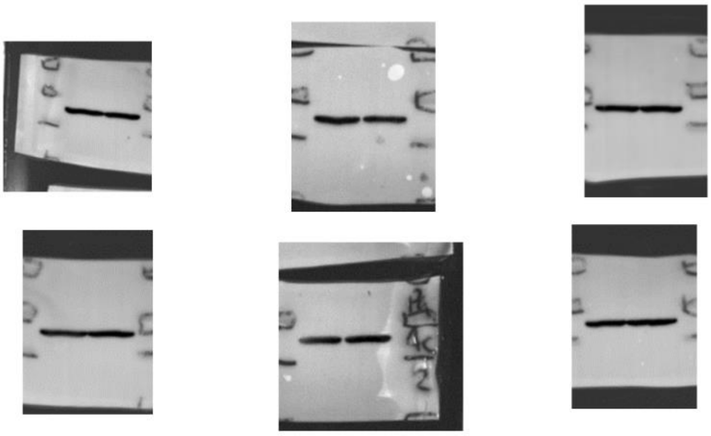

Eplerenone

Esaxerenone

Figure 7. Original electrophoresis gels and immunoblotting membranes.
